# Significance of kidney biopsy in autosomal dominant tubulointerstitial kidney disease-UMOD: is kidney biopsy truly nonspecific?

**DOI:** 10.1186/s12882-020-02169-x

**Published:** 2021-01-04

**Authors:** Tamehito Onoe, Satoshi Hara, Kazunori Yamada, Takeshi Zoshima, Ichiro Mizushima, Kiyoaki Ito, Takayasu Mori, Shoichiro Daimon, Hiroaki Muramoto, Maki Shimizu, Akira Iguchi, Akihiro Kuma, Yoshifumi Ubara, Michihiro Mitobe, Hiroaki Tsuruta, Nao Kishimoto, Junko Imura, Tadashi Konoshita, Mitsuhiro Kawano

**Affiliations:** 1 Division of Rheumatology, Department of Internal Medicine, Kanazawa University Graduate School of Medicine, Kanazawa, Ishikawa Japan; 2 Kanazawa Medical University, Department of Hematology and Immunology, Uchinada-Machi, Ishikawa Japan; 3 Department of Nephrology, Tokyo Medical and Dental University, Yushima, Tokyo, Japan; 4 Department of Nephrology, Daimon Clinic for Internal Medicine, Nephrology and Dialysis, Nonoichi, Ishikawa Japan; 5 Department of Internal Medicine, Japan Health Care Organization Kanazawa Hospital, Kanazawa, Ishikawa Japan; 6 Shimizu Children’s Clinic, Takamatsu, Kagawa Japan; 7 Department of Internal Medicine, Nagaoka Red Cross Hospital, Nagaoka, Niigata Japan; 8 University of Occupational and Environmental Health School of Medicine, Second Department of Internal Medicine, Kitakyusyu, Fukuoka Japan; 9 Nephrology Center and Okinaka Memorial Institute for Medical Research, Toranomon Hospital, Toranomon, Tokyo, Japan; 10 Department of Nephrology, Kameda Medical Center, Kamogawa, Chiba Japan; 11 Nagahama City Hospital, Department of Nephrology and Metabolism, Nagahama, Shiga Japan; 12 Osaka Saiseikai Izuo Hospital, Department of Nephrology, Osaka-city, Osaka Japan; 13 Imura Clinic, Department of Nephrology and Dialysis, Hakusan-city, Ishikawa Japan; 14 Third Department of Internal Medicine, University of Fukui Faculty of Medical Sciences, Eiheiji, Fukui Japan

**Keywords:** Autosomal dominant tubulointerstitial kidney disease, Kidney biopsy, Tamm-Horsfall protein, Uromodulin

## Abstract

**Background:**

Autosomal dominant tubulointerstitial kidney disease (ADTKD) is a rare hereditary disease caused by a variety of genetic mutations. Carriers of a mutation in the responsible genes are at risk of reaching end-stage kidney disease typically in middle age. The frequency of this disease is assumed to be underestimated because of a lack of disease-specific signs. Pathological findings obtained from kidney of uromodulin related ADTKD (ADTKD-UMOD) patients are regarded as non-specific and less-informative for its diagnosis. This research was undertaken to evaluate the significance of kidney biopsy in ADTKD-UMOD patients.

**Methods:**

Thirteen patients from 10 families with nine identified uromodulin (UMOD) gene mutations who underwent kidney biopsy in the past were studied. Their kidney tissues were stained with anti-UMOD antibody in addition to conventional methods such as PAS staining. When positive, the numbers of tubules with visible UMOD protein accumulations were calculated based on the total numbers of UMOD expressing tubules. Pathological findings such as tubulointerstitial fibrosis, atrophy, inflammation and glomerulosclerosis were also evaluated and analyzed.

**Results:**

Interstitial fibrosis and tubular atrophy were present in all 13 patients. Most atrophic tubules with thickening and lamellation of tubular basement membranes showed negative UMOD staining. In all but two patients with C94F mutations, massive accumulation of UMOD proteins was observed in the renal endoplasmic reticulum. UMOD accumulations were also detectable by PAS staining as polymorphic unstructured materials in the 11 patients at frequencies of 2.6–53.4%. 80.4% of the UMOD accumulations were surrounded by halos. The detection rate of UMOD accumulations positively correlated with eGFR. Glomerulosclerosis was detected in 11/13 patients, with a frequency of 20.0 to 61.1%, while no cystic dilatations of glomeruli were detected.

**Conclusions:**

Massively accumulated UMOD proteins in ADTKD-UMOD kidneys are detectable not only by immunostaining using anti-UMOD antibody but also by conventional methods such as PAS staining, although their detection is not easy. These findings can provide important clues to the diagnosis of ADTKD-UMOD. Kidney biopsy in ADTKD-UMOD may be more informative than assumed previously.

## Background

Autosomal dominant tubulointerstitial kidney disease (ADTKD) is a rare genetic disease, whose characteristics include progressive kidney injury, interstitial fibrosis and tubular atrophy. Uromodulin (UMOD), Mucin-1 (MUC1), renin (REN), Hepatocyte nuclear factor 1 beta (HNF1β) and alpha subunit of the endoplasmic reticular membrane translocon (SEC61A1) are the genes responsible for ADTKD [1, 2].

UMOD which encodes Tamm-Horsfall protein is the first identified and one of the most common genes to cause ADTKD [1, 3]. Autosomal dominant tubulointerstitial kidney disease caused by UMOD gene mutation (ADTKD-UMOD) used to be named medullary cystic kidney disease type2 (MCKD2; MIM 603860), glomerulocystic kidney disease (GCKD), familial juvenile hyperuricemic nephropathy (FJHN; MIM 162000) or uromodulin kidney disease (UKD) [1, 4]. However because cysts are not pathognomonic and may cause confusion, the new terminology ADTKD-UMOD has been proposed instead [1]. Besides common features of ADTKD such as autosomal dominant trait, progressive kidney injury and interstitial tubulopathy, early onset of hyperuricemia and gout are well-known characteristics of ADTKD-UMOD.

However most of the clinical signs of ADTKD such as decreased glomerular filtration rate (GFR) or hyperuricemia are very common in chronic kidney disease (CKD) of other etiologies too. This makes ADTKD a condition without disease-specific manifestations, and so difficult to diagnose.

Urinalysis of ADTKD patients usually shows a bland urinary sediment and no or little proteinuria which might delay early detection of ADTKD. Furthermore ADTKD is not a well-known disease even amongst nephrologists, and so a considerable number of ADTKD patients may reach end-stage kidney disease without an accurate diagnosis ever having been made.

Pathological findings of kidneys in ADTKD patients are reported to be non-specific, such as interstitial fibrosis, tubular atrophy, thickening and lamellation of tubular basement membranes with normal glomeruli or glomerulosclerosis. The role of renal biopsy in the process of ADTKD diagnosis is very limited. For example in single cases without positive family histories who are suspected of having ADTKD, renal biopsy serves to show tubulointerstitial nephritis and to exclude other renal diseases [5].

The mechanisms underlying kidney injury progression in ADTKD-UMOD are thought to be as follows. Abnormal UMOD protein encoded by mutated UMOD gene causes protein misfolding. These mutated UMOD proteins cannot exit the endoplasmic reticulum (ER) and so accumulate massively in ERs in the cytoplasm of TALH (thick ascending limb of Henle) cells. Excessively accumulated UMOD proteins trigger ER stress and tubulointerstitial inflammation and fibrosis.

The abundant accumulations of UMOD proteins are visible in kidney tissue immunostaining using anti UMOD antibody, and even more clearly by immunofluorescence staining [6]. In light microscopic observation of kidney biopsy samples, detection of abnormal UMOD accumulations in tubular cells is quite difficult. However some reports have shown visible UMOD accumulations in periodic-acid Schiff (PAS) or Masson trichrome staining in ADTKD-UMOD kidneys [6, 7]. Eosinophilic fluffy inclusions in TALH are reported to be one histologic feature of ADTKD-UMOD in the textbook of “Diagnostic pathology, kidney disease” [8]. Still the degree or frequencies of these findings in ADTKD-UMOD kidneys have yet to be fully determined. To better characterize these findings, the present study was undertaken to clarify the microscopic features of ADTKD-UMOD kidneys as well as to determine the role and significance of kidney biopsy in the diagnosis of ADTKD.

## Methods

Thirteen Japanese ADTKD patients from 10 families with 9 UMOD mutations who underwent kidney biopsies in the past were studied. The disease in most of them had been suspected from their clinical symptoms and extensive family histories of CKD and confirmed by genetic analysis [9–12]. The diagnosis of one patient without any family history (case #3) was made in our previous report whose purpose was to detect ADTDK-UMOD among 3787 patients who had undergone kidney biopsies in our affiliated facilities. Case #3 was one of 15 patients with renal insufficiency, hyperuricemia and normal urine tests without glomerular abnormalities under 50 years old extracted from the 3787 patients. Abnormal UMOD accumulation was detected in the kidney of case #3, and subsequently UMOD mutation was detected [13]. Another patient without a family history (case #9) was suspected to have ADTKD from the clinical history and kidney pathological findings which prompted genetic testing.

The severity of tubulointerstitial diseases is assessed by PAS and Masson trichrome staining. The degrees of interstitial fibrosis (ci) (0: 0–5%, 1: 6–25%, 2: 26–50%, 3: > 50%), tubular atrophy (ct) (0: none, 1: − 25%, 2: 26–50%, 3: > 50%), and interstitial inflammation (i) (0: − 10%, 1: 10–25%, 2: 26–50%, 3: > 50%)were scored 0–3 according to the Banff 2015 grading system [14]. The presence of cystic dilatation of bowman capsules and frequency of glomerulosclerosis were also assessed.

The presence and location of UMOD proteins in TALH epithelial cells were evaluated by immunofluorescence staining using anti-UMOD antibody (Santa Cruz Biotechnology, INC, CA, USA). The staining method followed the details outlined in a previous report [13]. To ascertain the intracellular location of UMOD protein accumulations, a kidney biopsy sample was stained simultaneously with various organelle markers in an ADTKD-UMOD patient with A247P mutation (case #1) in addition to anti-UMOD antibody. Anti-PDI (protein disulfide isomerase) antibody (Enzo Life Sciences, Inc., Farmingdale, USA) was used for ER staining, anti-Golgin97 antibody (Thermo Fisher Scientific, Waltham, MA, USA) for Golgi staining, anti-LAMP-1 (Lysosomal associated membrane protein-1) antibody (Abcam, Cambridge, UK) for lysosome staining, and anti-chromogranin c antibody (Abcam, Cambridge, UK) for secretory granule staining. Regarding all of the remaining ADTKD-UMOD patients, double staining with UMOD and PDI was performed.

To evaluate and compare the visibility of UMOD accumulations in ordinary staining in light-microscopy, we stained a series of thin-slice kidney tissues with hematoxylin and eosin (HE), PAS, periodic acid methenamine silver (PAM), and Masson trichrome in addition to anti-UMOD antibody in another ADTKD-UMOD patient with A247P mutation (case #2). In each staining method, the detection rate of accumulated UMOD was calculated based on the full field of obtained kidney tissue (Detection rate: numbers of tubules having visible abnormal UMOD accumulations in each staining/total numbers of tubules with UMOD accumulations detected by UMOD staining). Regarding all of the remaining ADTKD-UMOD patients with abnormal UMOD accumulations in immunofluorescence staining and four normal controls (a: 33 years, M, IgAGN, eGFR 61.0 ml/min, b: 65 years, F, IgAGN, eGFR 67.0 ml/min, c: 68 years, M, Myeloma kidney, eGFR 28.0 ml/min, d: 16 years, M, Minor abnormality, eGFR 147.7 ml/min), the detection rate in PAS staining was calculated based on the full field of obtained kidney tissue. (Detection rate: numbers of tubules having visible abnormal UMOD accumulations in PAS staining/total numbers of tubules with UMOD accumulations detected by UMOD staining). The sizes of UMOD accumulations and halos around them were assessed. The method of classification: if accumulated UMOD proteins were bigger than epithelial cell nuclei: L, Smaller than nuclei: S, no halo:-, halo smaller than nuclei: +, halo bigger than nuclei: ++.

Correlations between all parameters: eGFR, age, scores of interstitial fibrosis, tubular atrophy, interstitial inflammation, percentage of glomerulosclerosis and detection rate of abnormal UMOD in PAS staining were tested using the Spearman’s test. A *p* value < 0.05 was regarded as statistically significant.

## Results

The clinical features, kidney pathology and UMOD mutations of cases are summarized in Table 1. All of the detected variants were missense mutations. Two different missense mutations were detected in one patient (case #8). The pathogenicity prediction of detected variants was evaluated using analysis tools: Provean [15], Polyphen2 [16] and Mutation assessor [17]. All of the variants were predicted to be pathological. (Supplemental Table 1).

**Table 1:** Clinical presentation, renal pathology and UMOD mutations of ADTKD-UMOD patients

No	sex	FH	UMOD variant	Year at biopsy	Age at biopsy	Renal pathology	eGFR (ml/min)	sUA (mg/dl)	U-prot	U-ob	Gout	HT	Cysts	Age at RRT	
1	M	8	A247P	2007	22	TIN, NS	55.3	9	–	–	–	+	–	–	Family A Ref [13]
2	F	8	A247P	1981	36	TIN	26.9	8.4	–	–	–	+	–	73	Family A Ref[13]
3	M	0	A247P	2005	31	NS	53.9	7.8	–	–	+	–	–	–	Ref [13]
4	F	3	P173R	2011	56	TIN, NS	39.9	7.5	±	–	–	+	–	–	ref [9]
5	F	4	C135G	2012	31	TIN, NS	59.7	6.9	–	–	–	–	–	–	ref [11]
6	M	4	C306S	2013	17	TIN, NS	10.1	10.2	±	–	–	+	+	20	Family B
7	F	4	C306S	1994	28	TIN	ND	ND	ND	ND	ND	ND	+	47	Family B
8	F	4	C120S.W373C	2016	28	TIN	33.9	10.8	–	–	–	–	–	–	
9	F	0	L352Q	2018	41	TIN, NS	15.2	T	–	–	+	–	+	–	
10	M	3	C282Y	2017	44	TIN	18.7	T	±	–	+	+	ND	–	
11	F	4	C317G	1996	42	TIN, NS	30.2	8.5	–	–	–	–	–	60	
12	M	4	C94F	2013	14	TIN, NS	58.9	9.4	±	–	–	–	–	–	Family C ref. [10]
13	M	4	C94F	1993	26	TIN, NS	ND	ND	ND	ND	+	ND	–	35	Family C ref. [10]

*M* male, *F* female, *FH* numbers of families with CKD histories, *TIN: tubulointerstitial nephritis, NS:* nephrosclerosis*, HT* hypertension*, RRT* renal replacement therapy

Five of 13 cases (38.5%) reached end-stage kidney disease requiring renal replacement therapy at an average age of 47 years (respectively 73, 20, 47, 60 and 35 years). No patients had marked proteinuria or hematuria, 11/12 cases had hyperuricemia (91.7%), 4/11 cases (36.4%) had experienced gout, and 5/11 cases (45.5%) had hypertension. Small numbers of kidney cysts existed in 3/11 cases (27.3%).

**Fig. 1:**
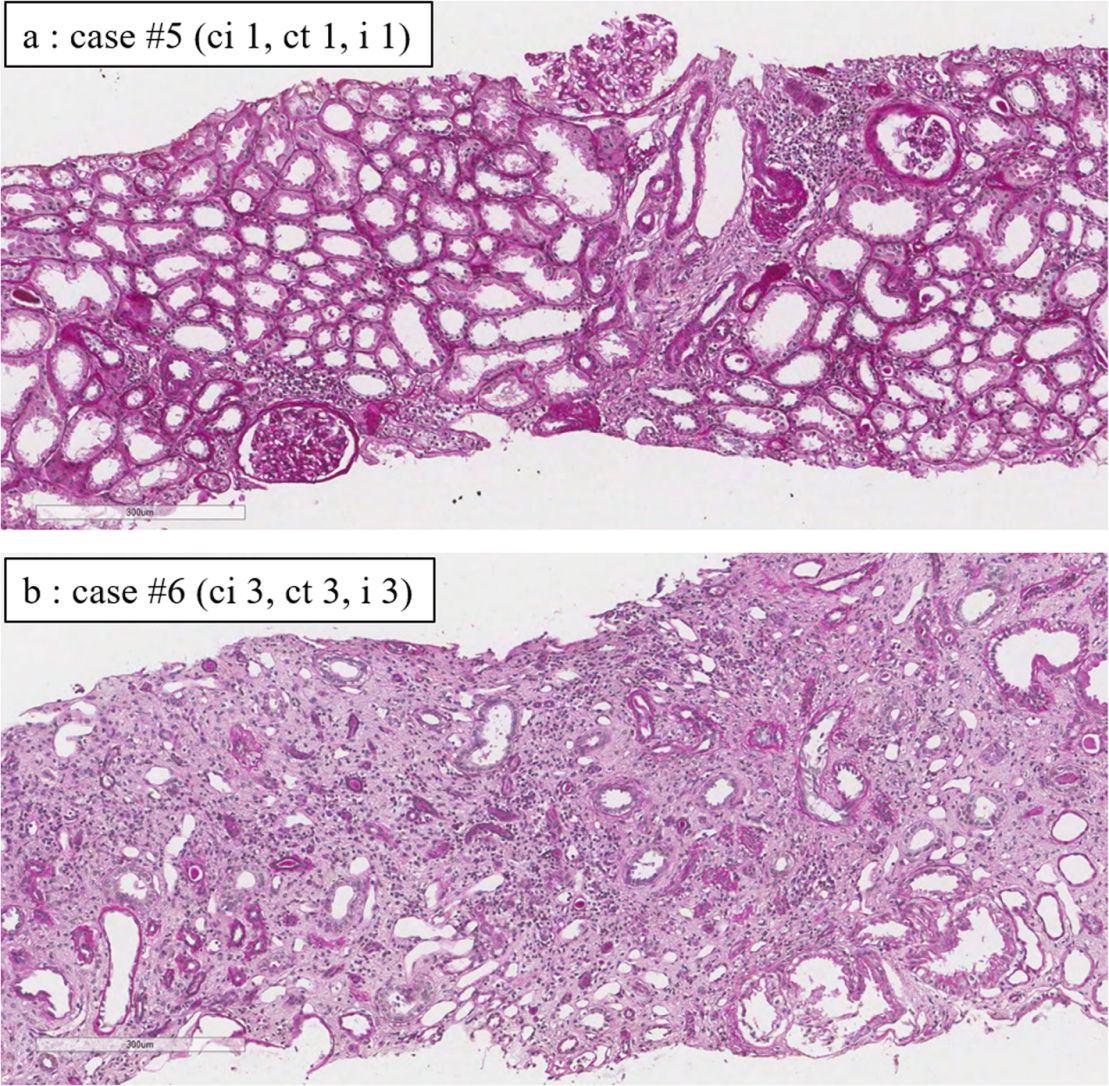
PAS staining of ADTKD-UMOD kidneys. Representative images of mild (a: case #5, ci 1, ct 1, i 1) and severe (b: case #6, ci 3, ct 3, i 3) cases were shown

The pathological diagnosis made from kidney biopsy of all the patients was tubulointerstitial nephritis and/or nephrosclerosis. Table 2 shows the scores of interstitial fibrosis, tubular atrophy, inflammation, and presence of glomerulosclerosis and cystic-dilated glomeruli in each ADTKD-UMOD patient. Immunofluorescence for complement and immunoglobulins was negative in all available patients. Representative images of mild and severe cases were shown in Fig. 1. In some patients, the numbers of glomeruli obtained by kidney biopsy were very small. Sclerotic glomeruli were detected in 11/13 patients with variable frequencies (17.8–61.1%). Cystic dilatation of glomeruli was not detected in any patient.

**Table 2:** Pathological characteristics of ADTKD-UMOD kidneys

	ci	ct	i	cystic glomerulus	screlotic/total glomerulus (%)
#1	1	1	1	0	4/17 (23.5%)
#2	2	2	1	0	0/2 (0%)
#3	1	2	1	0	6/18 (33.3%)
#4	2	2	0	0	11/18 (61.1%)
#5	1	1	1	0	5/28 (17.8%)
#6	3	3	3	0	3/6 (50%)
#7	1	1	0	0	0/8 (0%)
#8	2	1	1	0	1/2 (50%)
#9	1	2	1	0	7/26 (26.9%)
#10	1	1	1	0	2/6 (33%)
#11	2	1	1	0	3/15 (20%)
#12	3	2	2	0	6/17 (35.3%)
#13	3	1	1	0	6/12 (50%)

*ci* interstitial fibrosis (0: 0–5%, 1: 6–25%, 2: 26–50%, 3: > 50%)

*ct* tubular atrophy (0: none, 1: −25%, 2: 26–50%, 3: > 50%)

*i* interstitial inflammation (0: −10%, 1: 10–25%, 2: 26–50%, 3: > 50%)

**Fig. 2:**
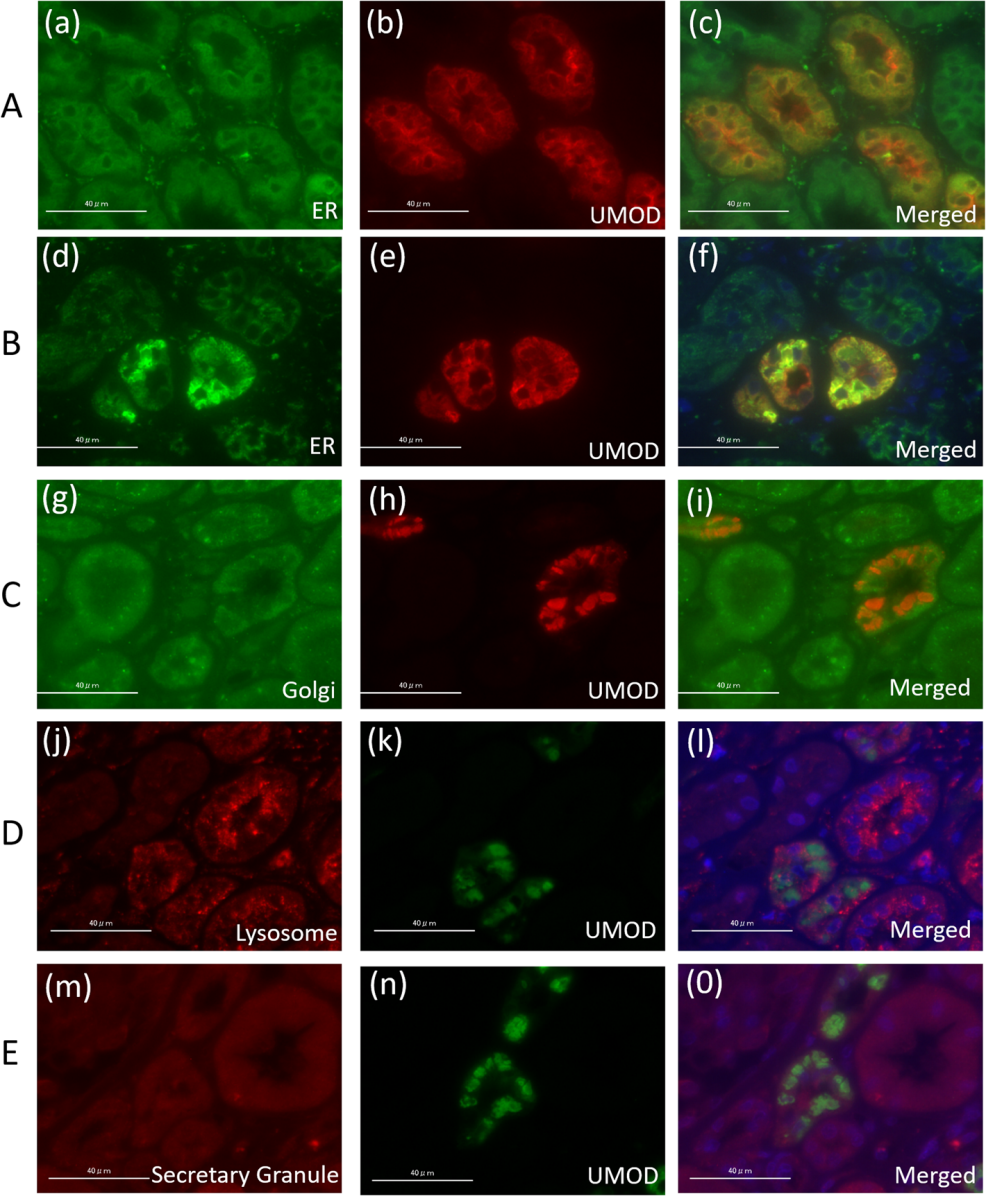
Locations of uromodulin proteins were evaluated by immunofluorescence staining using various organelle markers. UMOD proteins were simultaneously stained with anti-PDI antibody (ER marker, a, d), anti-golgin97 antibody (Golgi marker, g), anti- LAMP1 marker (lysosome marker j), and anti- chromogranin c antibody (secretory granule marker, m). c, f, i, l, o are merged images. **a**: Normal control kidney, fine UMOD proteins are located diffusely throughout the cytoplasm, and most intense signals are detected around the lumen of the tubules. **b**-**e**: Kidney of an ADTKD-UMOD patient (case #1) with A247P UMOD mutation. UMOD proteins accumulated irregularly inside the cytoplasm and concomitant increases of ER protein (PDI) were observed **f**. In these sections, golgin97, LAMP1 and chromogranin c neither co-localize with UMOD nor over-activate

In double-immunostaining using anti-UMOD antibody and various organelle markers in an ADTKD-UMOD patient kidney (case #1), accumulated UMOD proteins were merged with increased ER proteins but not with Golgi, lysosome or secretory granules (Fig. 2), meaning that mutated UMOD proteins are captured in ER and cannot proceed further.

**Fig. 3:**
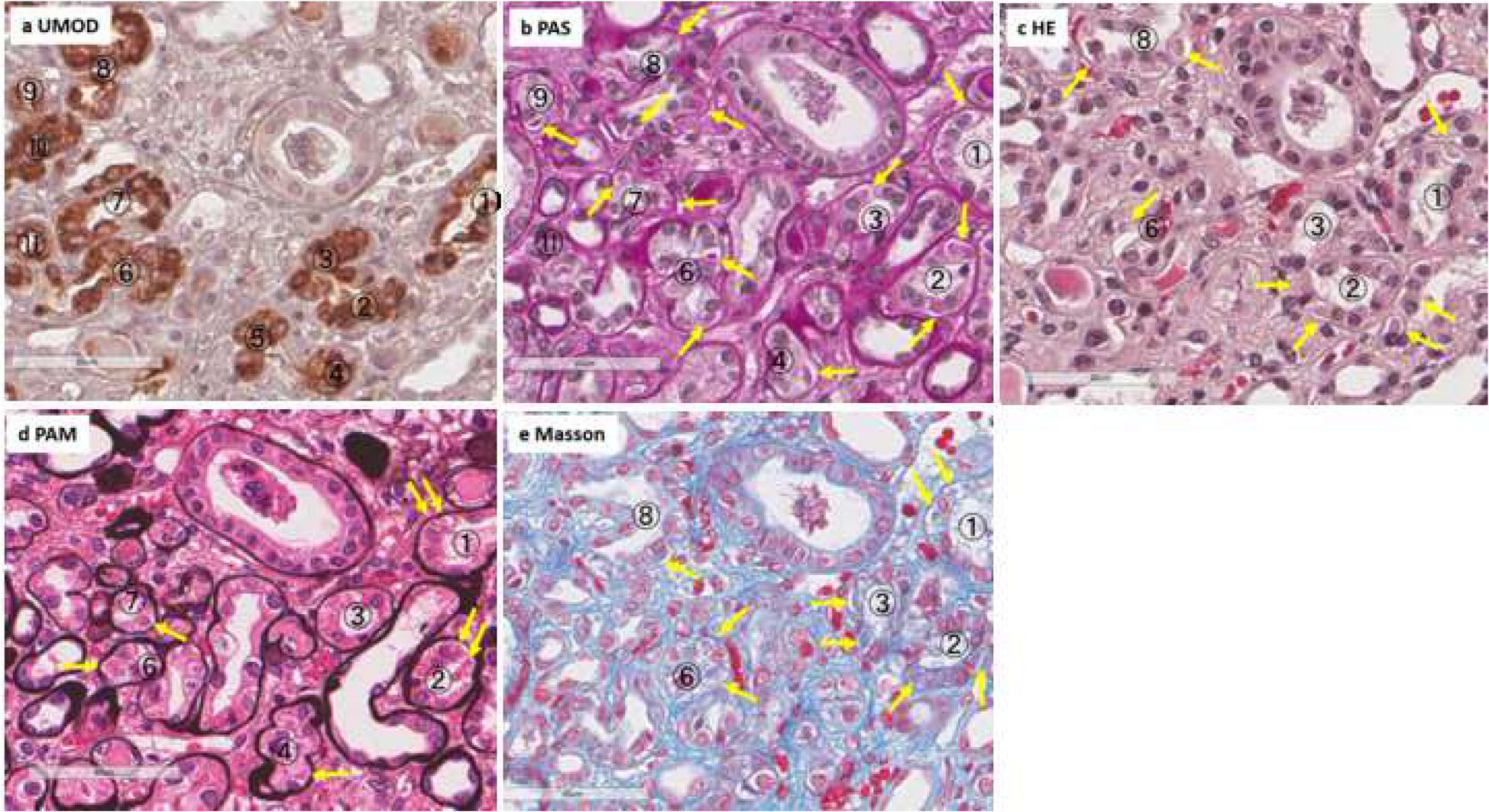
Series of the kidney specimens of an ADTKD-UMOD patient (case#2) with A247P were stained with PAS **b**, HE **c**, PAM **d**, Masson **e** in addition to anti-UMOD antibody **a**. Circled numbers indicate identical tubules which are positively stained with UMOD proteins. Arrows indicate visible UMOD protein accumulations in each staining. Accumulated UMOD proteins stain PAS positive, Trichrome blue, red in HE and PAM. The detection rate of each staining method is shown in Table 3 and PAS staining was revealed to be the most efficient to detect UMOD accumulations

In the series of light microscopic examinations using conventional staining methods (PAS, HE, PAM, Masson-trichrome) for another ADTKD-UMOD patient (case #2), UMOD accumulations were visible with all of the four staining methods and stained PAS positive, Trichrome blue, red in HE and PAM similar to previously reported findings [6, 7] (Fig. 3). The detection rates were 33.1% (PAS), 18.0% (Masson), 13.0% (HE) and 7.8% (PAM) respectively. Of them PAS staining was revealed to be the most efficient to detect UMOD accumulations (Table 3).

**Table 3:** Detection rate of uromodulin accumulations in each staining method (case #2)

	UMOD+ tubulus^a^	detection rate^b^
PAS	127	33.10%
HE	50	13%
PAM	30	7.80%
Masson	69	18%

a: numbers of tubules with detectable UMOD accumulations in each staining

b: a x100 / numbers of UMOD positive tubules in UMOD staining (384) these numbers are counted based on all field of obtained kidney tissue

**Fig. 4:**
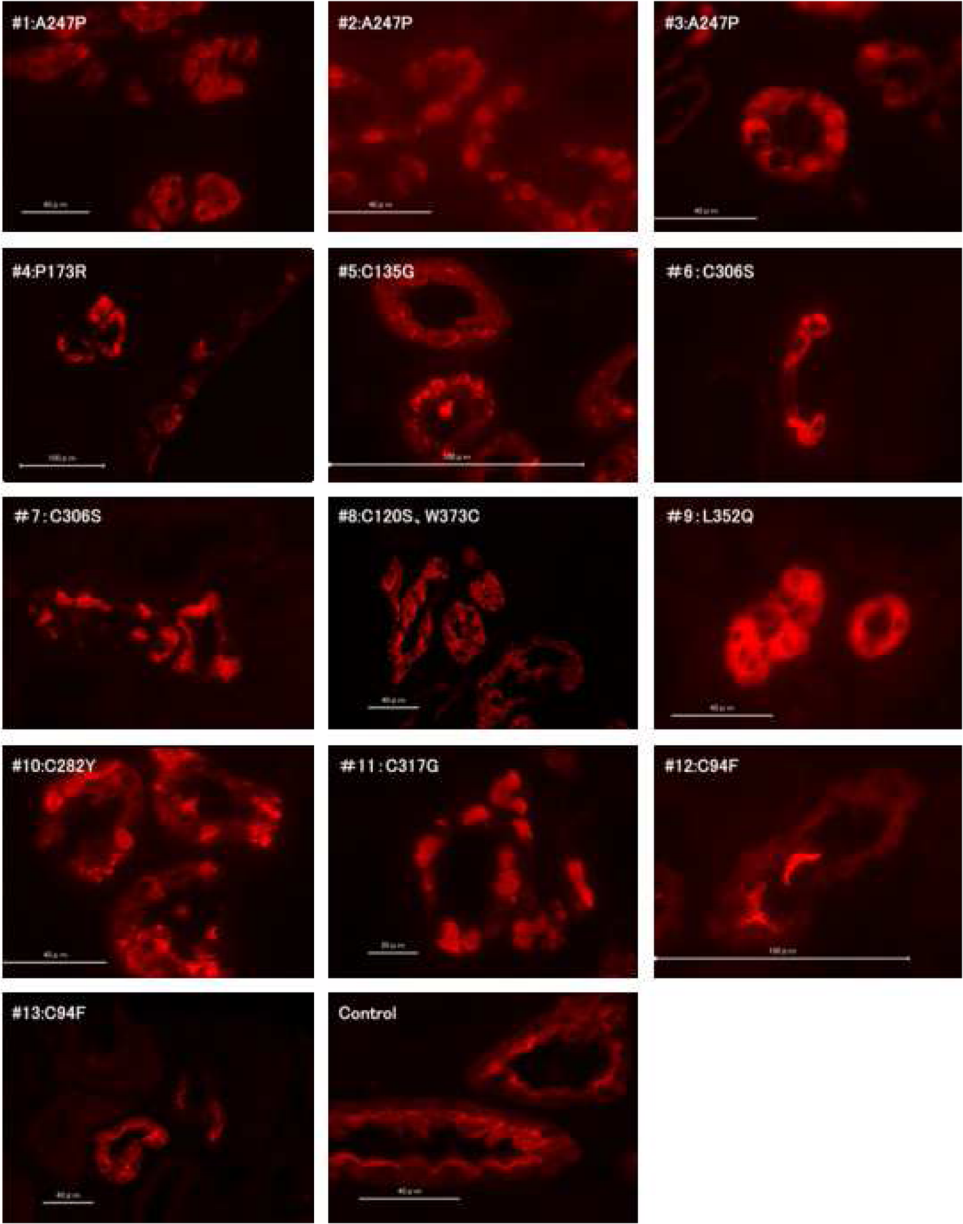
Immunofluorescence staining images using anti-UMOD Ab of ADTKD-UMOD patients cases #1-#13 and a control kidney. In all except two patients: a son and a father (cases #12 and #13) who share the same C94F UMOD mutations, UMOD proteins lost their original localization pattern, namely were scattered throughout the cytoplasm and collected around the tubular lumina. In their kidneys, UMOD proteins irregularly accumulate and make strong signals. In kidneys of ADTKD-UMOD patients with C94F mutations (#12 and #13), atrophy of UMOD positive TAL cells was marked. However the patterns of UMOD localization did not differ from those of normal kidney

In UMOD immunofluorescence staining, massive UMOD protein accumulations were detected in 11/13 cases (84.6%). Two patients: a father and a son with the same C94F mutations showed normal UMOD staining patterns throughout in highly atrophic tubules (Fig. 4).

**Fig. 5:**
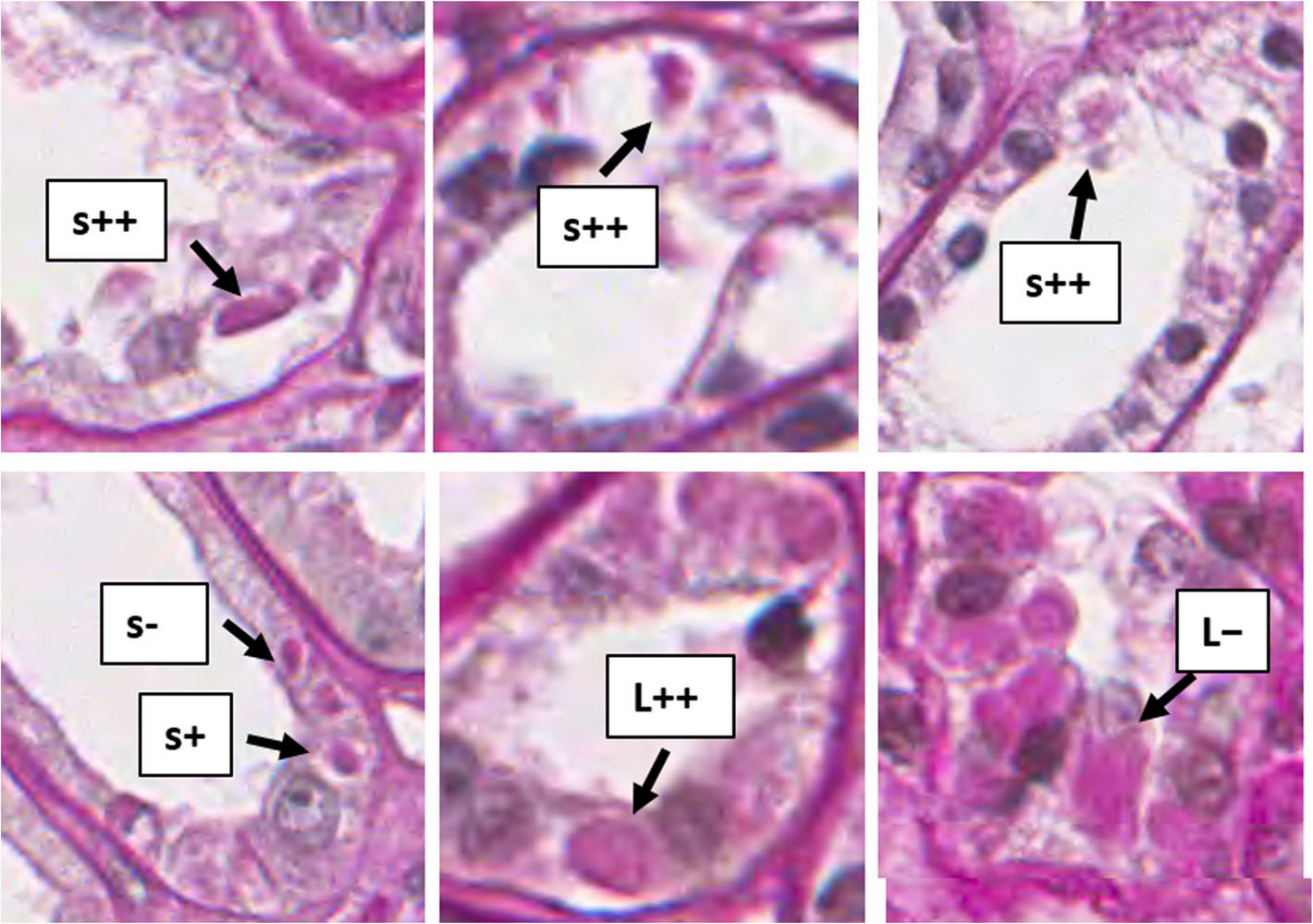
UMOD protein accumulation showed various appearances, sizes and densities in ADTKD-UMOD in PAS staining. L shows accumulated UMOD proteins bigger than epithelial cell nuclei, and S: smaller than nuclei. Aggregated UMOD proteins were PAS positive, irregularly shaped, and of various densities, with many of them having halos around them (no halo:-, halo smaller than nuclei: +, halo bigger than nuclei: ++)

**Fig. 6:**
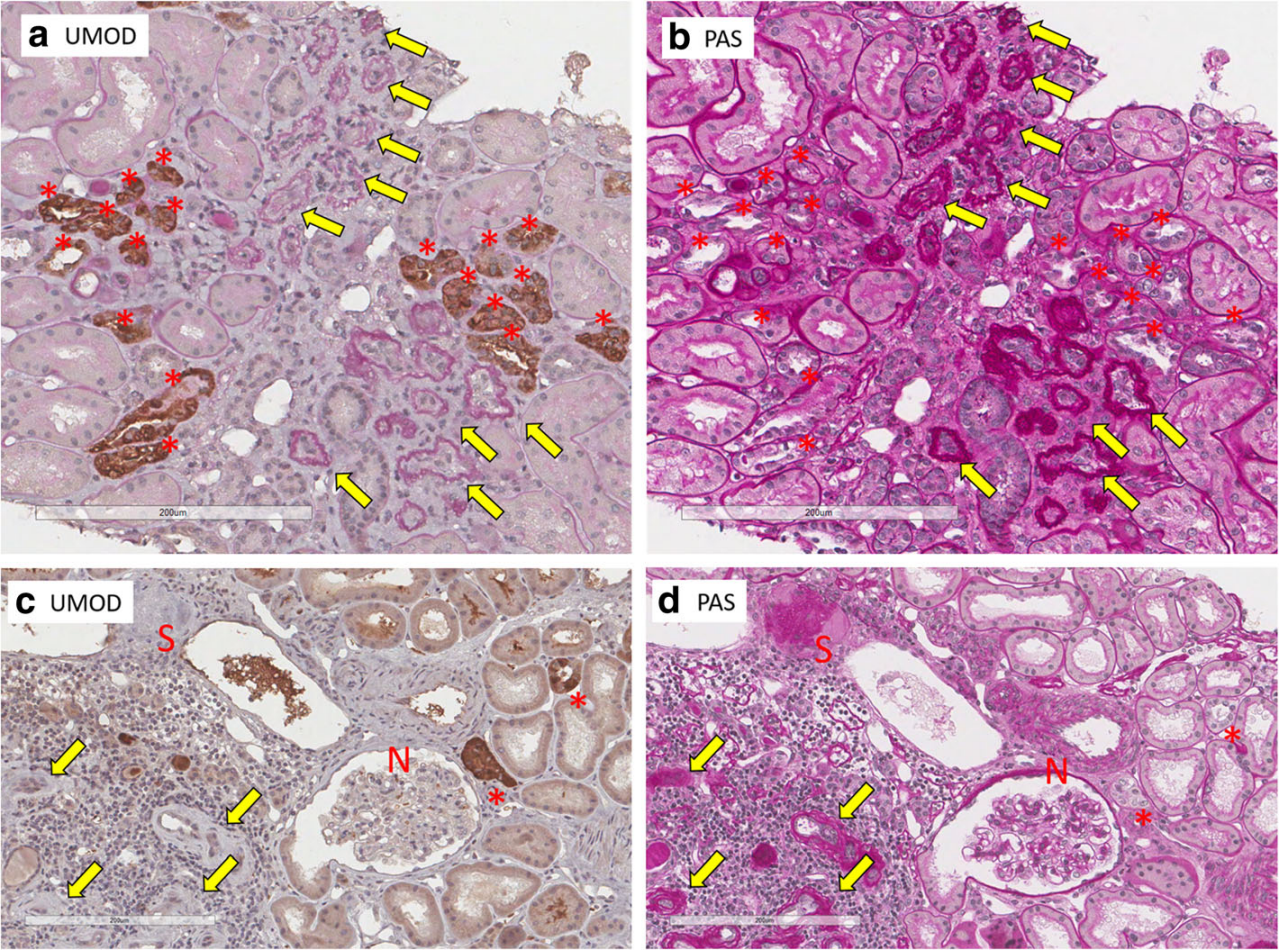
PAS staining **b**, **d** and UMOD immunostaining **a**, **c** of a series of ADTKD-UMOD patient kidneys (A and B: case #1, C and D: case #3). * Indicates uromodulin expressing TALH. Note most of the atrophic tubules with thickened and lamellation of tubular basement membranes (yellow allows) are UMOD negative. Prominent inflammatory cells infiltrate around a sclerotic glomerulus (S) and atrophic tubules. N indicates normal glomerulus

In all 11 cases with positive UMOD accumulations in fluorescence staining, UMOD accumulation was visible in PAS staining to variable degrees (2.6–57.1%) (Table 4). Each of the abnormally accumulated UMOD proteins detected in ADTKD-UMOD was PAS positive, irregularly shaped including round or oval-shaped, and showed various densities (Fig. 5). Many of them were surrounded by halos. Of tubules with UMOD accumulations, 34.7% had UMOD accumulations larger than the nuclei of epithelial cells and 80.4% had accumulated UMOD proteins with surrounding halos. 61.1% of them had halos bigger than the nuclei (Table 4). Interstitial inflammation tended to localize near atrophic tubules with thickened and lamellated basement membrane, although most of these atrophic tubules showed negative results in immunostaining using anti-UMOD antibody. Many sclerotic glomeruli were surrounded by infiltrates of inflammatory cells (Fig. 6). In the kidney of case #9, visible UMOD accumulations may have decreased because of the thickness of the specimen. In four control kidneys, PAS positive deposits were not detected in total 2345 UMOD positive TALH tubules (a: 548, b: 786, c:410, d: 601).

**Table 4:** The percentages and characteristics of tubules with visible UMOD accumulations in PAS staining

	total UMOD+ tubules	tubules with visible UMOD deposits					total	%^a^
		S-	S+	S++	L-	L++		
#1	126	19	19	6	8	20	72	**57.1%**
#2	384	11	49	18	7	42	127	**33.1%**
#3	564	37	92	36	32	92	289	**51.1%**
#4	302	9	60	42	20	35	166	**53.4%**
#5	913	8	62	102	3	34	209	**22.9%**
#6	26	0	1	1	0	1	3	**11.5%**
#7	41	1	4	2	1	3	11	**26.8%**
#8	78	21	6	0	4	1	32	**41.0%**
#9	38	0	0	0	0	1	1	**2.6%**
#10	123	1	5	6	2	6	20	**16.2%**
#11	176	9	20	16	6	36	87	**49.4%**
**sum**	**2733**	**116**	**318**	**229**	**83**	**270**	**1016**	37.2%

*S* detected UMOD aggregation inside tubules was smaller than epithelial cell nuclei, *L* bigger than nuclei

++ halo around UMOD bigger than epithelial cell nuclei, *+* smaller than nuclei, *−* no halo

^a^: numbers of tubules with visible UMOD accumulations in PAS staining/ total numbers of UMOD positive tubules in UMOD staining × 100

Statistical analysis showed the detection rate of UMOD accumulations to positively correlate with eGFR, while the score of interstitial inflammation negatively correlated with age (Table 5).

**Table 5:** Spearman’s rank correlation coefficient scores and significance value for each clinical and pathological parameter

		eGFR	Age	ci	ct	i	Glomerulosclerosis%	Detection rate%
eGFR	*ρ*	1.000	−0.296	−0.162	−0.352	−0.162	0.009	.709^*^
	*P*		0.377	0.635	0.289	0.634	0.979	0.022
Age	*ρ*		1.000	−0.394	−0.074	−.571^*^	− 0.188	0.032
	*P*			0.183	0.809	0.041	0.538	0.926
ci	*ρ*			1.000	0.339	0.477	0.504	0.051
	*P*				0.258	0.099	0.079	0.882
ct	*ρ*				1.000	0.386	0.102	−0.204
	*P*					0.193	0.739	0.548
i	*ρ*					1.000	0.218	−0.383
	*P*						0.475	0.245
Glomerulosclerosis%	*ρ*						1.000	0.037
	*P*							0.915
Detection rate%	*ρ*							1.000
	*P*							

* Correlation is significant at the 0.05 level (2-tailed), *ρ* correlation coefficient, *P p*-value

*ci* interstitial fibrosis, *ct* tubular atrophy, *i* inflammation

Glomerulosclerosis, *%* percentage of glomerulosclerosis, Detection rate%: detection rate of abnormal UMOD

## Discussion

Pathological characteristics of kidneys in 13 Japanese ADTKD-UMOD patients with identified UMOD mutations were evaluated. This is the first study to systematically analyze the pathology of multiple ADTKD-UMOD patients. In 11/13 patients, abnormal UMOD accumulations were detected both in immunostaining using anti-UMOD antibody and in PAS staining. It is possible that aggregated UMOD deposits are more detectable in the presence of higher GFRs suggesting that the value of kidney biopsy in ADTKD-UMOD is greater with higher GFRs. These findings may help to make the diagnosis of ADTKD-UMOD. When encountering a youngish patient suspected of having ADTKD with renal insufficiency and hyperuricemia without urinalysis abnormalities, close observation of kidney tissue obtained by biopsy would be a good option for further diagnosis when a family history of CKD is absent or unclear.

In 1978, Zager et al. [18] and Resnick et al. [19] showed interstitial deposition of PAS positive Tamm-Horsfall protein in MCKD kidney long before Hart et al. [20] reported that mutation of UMOD gene which codes Tamm-Horsfall protein is responsible for both FJHN and MCKD type2 in 2002. In 2003, Rampoldi et al. [4] found that UMOD protein formed globular masses within the cytoplasm of ADTKD-UMOD kidneys in immunopathology using anti-UMOD antibodies and confirmed these intra-cytoplasmic heaps by electron microscopy to be fibrillary material. Nasr et al. [7] and Christiansen et al. [6] reported abnormal UMOD accumulations as PAS-positive, trichrome blue intracytoplasmic inclusions in light microscopic observation of kidneys in independent ADTKD-UMOD patients respectively.

The pathological findings of ADTKD have been reported to be those of non-specific interstitial tubulopathy. Ekisi et al. [5] reported renal fibrosis to be a common feature of ADTKD in pathological investigations of 14 ADTKD patients including one with UMOD mutation. Ayasreh et al. [21] reported the clinical presentations of 131 Spanish ADTKD patients from 56 families, in 21 members of which including 2 ADTKD-UMOD patients kidney biopsy was performed. Their common pathological findings were tubular atrophy and interstitial fibrosis. This means that it is difficult to assume a diagnosis of ADTKD relying on kidney pathology alone.

In the 2015 KDIGO consensus report, K-U Echardt et al. [1] reported that an established diagnosis of ADTKD is made either by ①demonstration of a mutation in one of the responsible genes in an affected individual or at least one family member. ② presence of a family history compatible with autosomal dominant inheritance of CKD, consistent clinical characteristics and compatible histology in at least one affected family member. They also mentioned that it is not possible to make a definitive diagnosis by renal biopsy alone.

However, the possibility of ADTKD-UMOD is not excluded even when a family history of CKD is absent or unclear. Bolle et al. [22] reported that about 10% of ADTKD-UMOD patients have de novo mutations. It is difficult to affirm that a detected UMOD gene mutation is truly deleterious in any given patient in the absence of a convincing family history even if the mutation is predicted to be highly pathological in analysis software. But if abnormal UMOD protein accumulation is observed in the kidney of a patient with an ambiguous UMOD mutation, it can be one functional piece of evidence of the deleteriousness of the UMOD mutation, and thereby support the genetic diagnosis of ADTKD-UMOD.

Intracytoplasmic PAS positive deposits may be a pathological clue to suspect ADTKD-UMOD. However caution is needed. Crystalline inclusions of light chain proximal tubulopathy are also PAS positive deposits detected inside the cytosol of proximal tubular cells [23]. Blebbed and sloughed tubular epithelial cells resulting from acute tubular injury [24] or protein reabsorption droplets [25] predominantly detected in proximal tubules resemble similar PAS positive deposits. UMOD accumulations in ADTKD-UMOD are exclusively located in TALH in distal tubules and many of them (80.4%) have halos around them, making their location and the appearance of deposits important clues to their detection. Careful observation of distal tubules is necessary in patients suspected of having ADTKD-UMOD.

In the present study, we could not find abnormal UMOD accumulations immunohistochemically in the kidneys of the father and son who have C94F mutation in common with clinical courses of typical ADTKD-UMOD. This result was unexpected because the transgenic mice which have the identical mouse mutation manifest UMOD accumulations in their kidneys and develop kidney disease which resembles ADTKD [26]. This implies that another mechanism leading to kidney impairment exists other than ER stress reaction caused by abnormal UMOD accumulation in ADTKD-UMOD. Interestingly, most of the atrophic tubules in ADTKD-UMOD kidneys observed in the present study were not UMOD positive. El-Achkar et al. reported that necrotic tubules arising following ischemia reperfusion injury in UMOD knock-out mice are predominantly S3 segments of proximal tubules, which means a direct cross talk between different tubular segments may exist in a UMOD dependent manner [27]. Further investigations are needed to prove whether similar phenomena are present in human ADTKD-UMOD patients.

Our study had the following limitations. First, the analyzed case numbers may not have been sufficient. Second, electron microscopic observations were not performed in our patients. Third, we could not explain why the patients with C94F UMOD mutations did not have abnormal UMOD accumulations and why most of the atrophic tubules in ADTKD-UMOD were negative for UMOD staining. Further investigations will be needed to unravel these issues in the future.

## Conclusions

This study showed that aggregated UMOD accumulations are detectable in 85% of ADTKD-UMOD patients using PAS staining light microscopy examinations, demonstrating that pathological examinations of ADTKD-UMOD kidneys provide more useful information than previously reported.

## Supplementary Information

**Additional file 1 **: **Supplemental table 1**. pathogenicity prediction of each UMOD variant

## Data Availability

The datasets supporting the conclusions of this article are included within the article and its additional file.

## Abbreviations

ADTKD: Autosomal dominant tubulointerstitial kidney disease
UMOD: Uromodulin
MUC-1: Mucin-1
REN: Renin
HNF1β: Hepatocyte nuclear factor 1 beta
SEC61A1: Alpha subunit of the endoplasmic reticular membrane translocon 1 beta
ADTKD-UMOD: Uromodulin related autosomal dominant tubulointerstitial kidney disease
MCKD2: Medullary cystic kidney disease type 2
GCKD: Glomerulocystic kidney disease
FJHN: Familial juvenile hyperuricemic nephropathy
UKD: Uromodulin kidney disease
GFR: Glomerular filtration rate
CKD: Chronic kidney disease
ER: Endoplasmic reticulum
TALH: Thick ascending limb of Henle
PDI: Protein disulfide isomerase
LAMP-1: Lysosomal associated membrane protein-1
