# Supplementary material for: Significance of kidney biopsy in autosomal dominant tubulointerstitial kidney disease-UMOD: is kidney biopsy truly nonspecific?
Source: BMC Nephrol. 2021 Jan 4;22:1. doi: 10.1186/s12882-020-02169-x (PMC7784305; doi:10.1186/s12882-020-02169-x)
Supplement: Supplementary file 1 — Additional file 1 : Supplemental table 1. pathogenicity prediction of each UMOD variant [file 12882_2020_2169_MOESM1_ESM.docx]

Supplemental table 1　 pathogenicity prediction of each UMOD variant

| **UMOD variant** | **Provean** | **Mutation assessor** | **Polyphen2** |
| --- | --- | --- | --- |
|  | score | FI score | Hum Div |
| C135G | -5.622 | 4.665 | 1 |
|  | deleterious | high | probably damaging |
| A247P | -3.285 | 3.3 | 1 |
|  | deleterious | medium | probably damaging |
| P173R | -6.575 | 3.65 | 1 |
|  | deleterious | high | probably damaging |
| C306S | -7.58 | 3.32 | 1 |
|  | deleterious | medium | probably damaging |
| C120S | -4.689 | 4.105 | 1 |
|  | deleterious | high | probably damaging |
| W373C | -6.968 | 2.375 | 1 |
|  | deleterious | medium | probably damaging |
| C94F | -4.035 | 4.575 | 1 |
|  | deleterious | high | probably damaging |
| L352Q | -4.411 | 2.775 | 1 |
|  | deleterious | medium | probably damaging |
| C282Y | -8.044 | 3.85 | 1 |
|  | deleterious | high | probably damaging |
| C317G | -9.638 | 3.34 | 1 |
|  | deleterious | medium | probably damaging |
